# Artificial intelligence in orthopedics: current applications, challenges, and future directions

**DOI:** 10.1186/s43019-026-00317-5

**Published:** 2026-04-03

**Authors:** Sang Yoon Kim, Byung Sun Choi, Hyuk-Soo Han, Du Hyun Ro

**Affiliations:** 1 Department of Orthopaedics, Seoul National University Hospital, Seoul, Korea, Republic of; 2 CONNECTEVE Co., Ltd, Seoul, South Korea; 3 Institute of Convergence Medicine With Innovative Technology, Seoul National University Hospital, Seoul, South Korea; 4 Department of Orthopaedic Surgery, Seoul National University College of Medicine, Seoul, South Korea; 5 Healthcare AI Research Institute, Seoul National University Hospital, Seoul, South Korea

**Keywords:** Artificial intelligence, Orthopedics, Machine learning, Deep learning, Large language models, Musculoskeletal imaging, External validation, Workflow integration, Clinical translation

## Abstract

**Background:**

Artificial intelligence research in orthopedics has grown rapidly, yet a substantial gap remains between technical development and clinical translation. This narrative review summarizes current applications of artificial intelligence in orthopedic practice and highlights barriers to implementation.

**Main body:**

Current work converges on three domains: machine learning for structured perioperative risk prediction, deep learning for standardized musculoskeletal imaging, and large language models for workflow and decision support. Applications such as automated fracture detection, Kellgren–Lawrence grading for osteoarthritis, and transfusion risk modeling are approaching clinical maturity. However, routine adoption is limited by algorithmic opacity, performance degradation in new clinical environments, and poor fit within existing workflows. We argue that progress should shift from increasing model complexity toward rigorous evaluation, including external validation on independent cohorts. In addition, probability calibration and uncertainty estimation are important for trustworthy risk communication. Future directions may include multimodal “digital twin” approaches that integrate electronic medical records, imaging phenotypes, and intraoperative data into patient-specific trajectories.

**Conclusions:**

The clinical impact of artificial intelligence in orthopedics will depend on life-cycle governance and demonstrated net benefit, prioritizing reliability and implementation science over retrospective benchmark performance.

## Background

Artificial intelligence (AI) has transitioned from a computational curiosity to a ubiquitous presence in modern society, reshaping industries from finance to autonomous transportation. In medicine, this expansion is equally profound, with active research spanning radiology, internal medicine, surgery, and pathology [1]. Orthopedic surgery sits at a compelling intersection of acute clinical need and vast data availability. Population aging and the rising global burden of musculoskeletal disease have driven unprecedented demand for joint-preserving and reconstructive procedures, while regional disparities in specialist availability and personnel shortages strain existing systems. Against this backdrop, AI is positioned not merely as a tool for automation, but as a means to enhance diagnostic precision, standardize complex measurements, and streamline perioperative planning without proportionally increasing clinician workload.

The exponential trajectory of orthopedic AI research is quantifiable. In a bibliometric analysis focused on AI in arthroplasty, Li and colleagues identified 867 articles and reviews published between 2000 and 2021 and reported exponential growth in annual output over the past two decades [2].

However, this research surge has not translated uniformly to the bedside. While certain models—such as perioperative risk scores or automated radiographic measurements—are nearing routine use, others, including multimodal systems and large language models (LLMs), remain experimental [3]. For many surgeons, the field can feel simultaneously overhyped and opaque, characterized by impressive metrics but uncertain clinical value.

To provide a pragmatic roadmap for clinicians, we organize orthopedic AI into three converging strands—machine learning (ML) for structured risk prediction, deep learning (DL) for standardized image analysis, and large language models (LLMs) for workflow and decision augmentation—each constrained by shared bottlenecks, including domain shift, algorithmic opacity, and incomplete workflow integration. This review takes the position that the next phase of orthopedic AI will depend less on inventing ever more complex models and more on how we design, evaluate, and embed them. From this perspective, three questions become central: which orthopedic tasks are sufficiently mature for staged clinical deployment; how shared bottlenecks—data, generalizability, and explainability—can be overcome; and which design principles distinguish tools that provide net clinical benefit from those that remain academic prototypes. This article is a narrative review intended for orthopedic surgeons and trainees, designed to provide a clinician-facing framework for translation rather than an exhaustive catalog of algorithms.

## Data ecosystems and methods in orthopedic AI

Orthopedic AI has emerged at the intersection of three enabling trends: the digitization of clinical and imaging data, dramatic increases in computing power, and advances in machine learning algorithms [4]. In practice, orthopedic AI is built on heterogeneous data streams generated during routine care, processed by different modeling paradigms—ML, DL, and LLMs—that must be chosen and evaluated with attention to task, data, and deployment context.

*Literature scope and selection approach.* This narrative review used a purposive selection strategy rather than a systematic evidence synthesis. Studies were prioritized if they met at least one of the following: (1) publication primarily between 2020 and 2025 with external validation across independent cohorts or institutions; (2) demonstration of clinical workflow integration and/or prospective evaluation (including silent-mode deployment); or (3) methodological relevance to emerging multimodal or implementation-focused orthopedic AI [5]. Earlier landmark studies were selectively included when necessary to provide historical or conceptual context and to avoid over-reliance on single-institution experiences. Large language model assistance was used for language editing and clarity only; all content was written, verified, and approved by the authors.

### Clinical and imaging data sources for orthopedic AI

The “fuel” for orthopedic AI consists of several broad classes of data already embedded in daily practice. A first pillar is structured clinical information captured in electronic medical records (EMRs) and administrative databases. Demographics, comorbidities, laboratory values, medications, and procedure codes can be encoded as tabular variables and have become the backbone of perioperative and longitudinal outcome prediction models, such as those targeting transfusion or 30-day readmission after arthroplasty [6, 7]. These data are routinely available, relatively standardized, and well-suited to conventional ML approaches.

A second pillar is imaging data archived in Picture Archiving and Communication Systems (PACS). Plain radiographs, computed tomography (CT), and magnetic resonance imaging (MRI) are indispensable in orthopedics: radiographs underpin fracture diagnosis and alignment analysis, while MRI provides high-resolution visualization of cartilage, menisci, and ligaments [8, 9]. PACS facilitates large-scale curation of anonymized Digital Imaging and Communications in Medicine (DICOM) files, enabling convolutional neural network (CNN)-based models for fracture classification, osteoarthritis (OA) grading, and automated measurement of alignment and morphology [10–12]. Representative applications include deep learning systems for hip-fracture detection and classification and automated radiographic knee OA grading [12–15].

Textual documents form a third, historically underused source. Operative notes, radiology reports, clinic letters, and discharge summaries encode nuanced clinical reasoning and patient preferences but have been difficult to exploit because they are unstructured. Recent advances in LLMs now offer practical methods to extract structured information from these narratives and generate drafts of clinician-facing documentation under supervision [16–18].

Beyond EMR tables, images, and text, registry and claims data contribute longitudinal perspective. National joint registries, hospital-based arthroplasty databases, and insurance claims track implant survival, complications, revision procedures, and resource utilization over years to decades. When linked with EMR and imaging data, these sources enable long-term risk prediction and health-economic analyses beyond individual institutions and single episodes of care [19].

Finally, newer data streams are beginning to enter orthopedic AI. High-resolution physiologic and anesthetic monitoring data, continuous telemetry, operating-room video, and wearable-device outputs are being captured more systematically and offer opportunities for finer-grained perioperative risk modeling and objective monitoring of rehabilitation trajectories [4, 20].

**Fig. 1:**
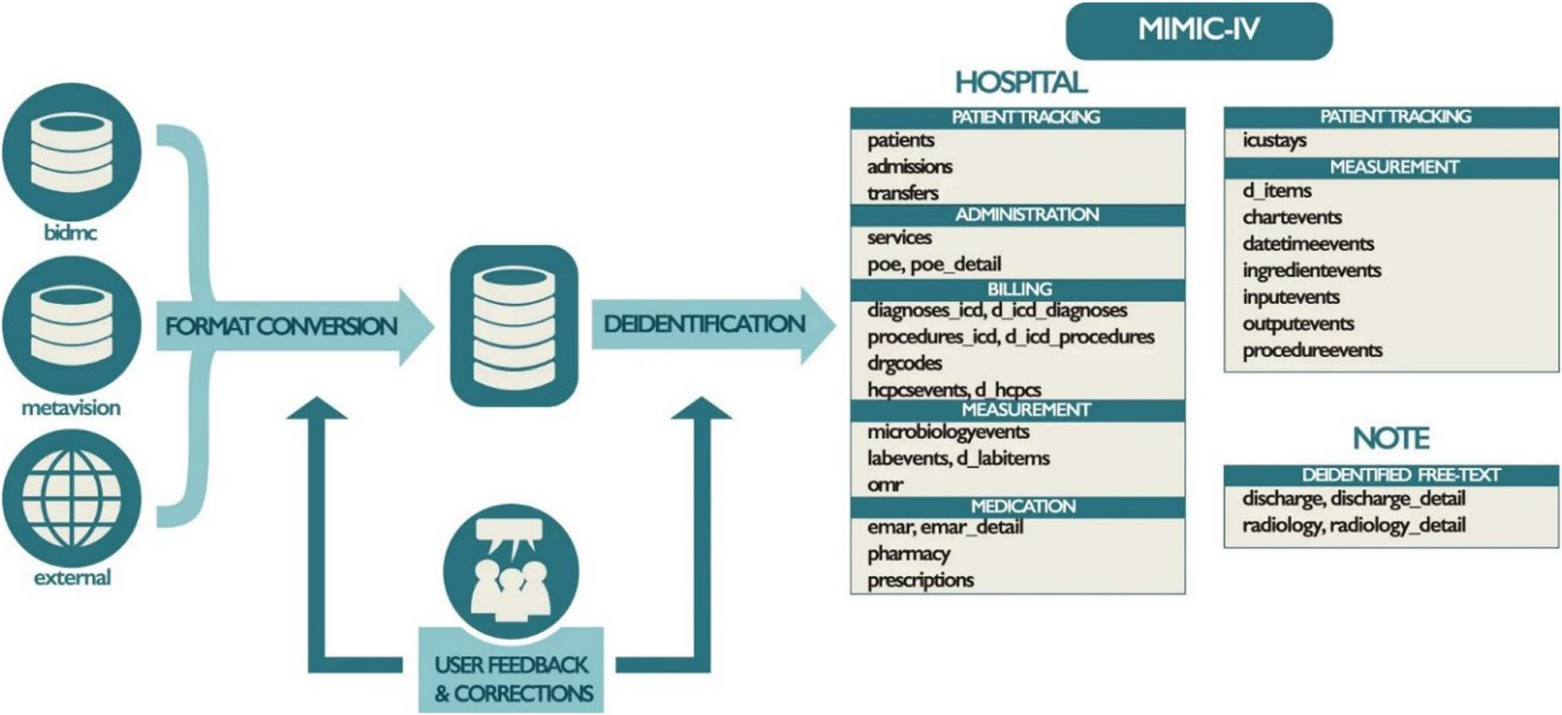
Structure and content of the MIMIC-IV database and typical AI development workflow. The diagram summarizes linked data domains—electronic medical records (EMR), intensive-care waveforms and vital signs, laboratory values, medications and procedures, and imaging (e.g., chest radiographs)—and illustrates how deidentified records flow into cohort curation, feature/label definition, model training/validation, and prospective evaluation. EMR indicates electronic medical record; ICU, intensive care unit. This workflow underscores the implementation science and governance required for clinical translation. Adapted from Johnson et al. [21] under the Creative Commons Attribution 4.0 International License (CC BY 4.0); changes were made

The intensive-care dataset Medical Information Mart for Intensive Care IV (MIMIC-IV), developed at Beth Israel Deaconess Medical Center, illustrates the power of curated, deidentified EMR data for AI research at scale [21]. Inspired by this paradigm, multi-hospital consortia have begun building large-scale datasets that incorporate imaging and high-resolution biosignals. Although not orthopedic-specific, these initiatives exemplify the infrastructure, standardization, and governance models needed for large, multicenter clinical AI efforts [20]. In musculoskeletal care, analogous efforts include OA cohorts, arthroplasty registries, and multicenter imaging initiatives, creating a data ecosystem that supports the applications described in Patient outcome prediction–Large language models (LLMs) section. The typical workflow for integrating heterogeneous clinical data streams into a validated AI model is illustrated in Fig. 1.

### Choosing the right modeling paradigm: ML, DL, and LLMs

Although AI is often used as a single umbrella term, different modeling paradigms are better suited to different orthopedic tasks. ML, in its classical sense, refers to algorithms that learn patterns from structured data to make predictions or classifications. Common methods include regularized logistic regression, decision trees, random forests, and gradient-boosted machines. These models are well matched to tabular EMR and registry variables and have been widely applied to perioperative risk stratification and longitudinal prediction—such as transfusion, NSAID-related complications, or 30-day readmission after arthroplasty—because they can be parsimonious, easier to calibrate, and amenable to feature-importance analysis [7, 22].

DL encompasses multilayer neural networks capable of learning complex, hierarchical representations from high-dimensional inputs such as images, waveforms, or sequences [4, 9]. CNNs dominate image-based tasks and have become the standard for fracture detection, OA grading, and automated alignment measurement in musculoskeletal imaging [10, 13]. When trained on sufficient data, CNNs consistently outperform traditional handcrafted-feature approaches in both accuracy and robustness. Techniques such as transfer learning, contrastive/self-supervised pretraining, and data augmentation are frequently employed to address limited labeled datasets and to improve generalization across scanners and patient populations [23–25]. Key technical and operational distinctions between ML and DL are summarized in Table 1.

**Table 1:** Machine learning versus deep learning in orthopedic research

Dimension	Machine learning (ML)	Deep learning (DL)
Core objective	Predict or classify using explicitly defined, structured clinical features	Learn hierarchical representations directly from high-dimensional raw data
Typical inputs	Structured EMR data (age, body mass index (BMI), comorbidities), lab values, and registry variables	Radiographs, CT, MRI, ultrasound, and high-frequency sensor/wearable data
Feature engineering	Manual: requires expert-driven feature selection and clinical preprocessing	Automated: CNN or Transformer architectures learn optimal features end-to-end
Data scale requirements	Adequate performance achievable with modest, institutional-sized cohorts	Requires large-scale labeled datasets; often mitigated by transfer learning
Computational resources	Low-to-moderate; typically executable on standard central processing unit (CPU)–based systems	High; necessitates dedicated graphics processing unit (GPU) / tensor processing unit (TPU) resources and significant memory
Interpretability	Inherent: clearer logic via Shapley additive explanations (SHAP), feature importance, or decision rules	Mediated: perceived as “black-box”; requires explainable AI (XAI) methods (e.g., gradient-weighted class activation mapping [Grad-CAM], attention maps)
Generalization	Sensitive to feature-level shifts; stabilized via regularization/selection	Vulnerable to domain shift (scanners/sites); requires robust external validation
Representative algorithms	Logistic regression, Random Forest, extreme gradient boosting (XGBoost), Support Vector Machines	CNNs (ResNet, EfficientNet), U-shaped network (U-Net), Vision Transformers (ViT)
Strengths	Fast development; high transparency; straightforward risk calibration	State-of-the-art performance on vision tasks; captures subtle spatial patterns
Orthopedic exemplars	Transfusion prediction (AUROC≈0.84); Postoperative readmission risk modeling	Hip fracture classification (> 95% accuracy); Automated KL grading; hip–knee–ankle angle (HKAA)/medial proximal tibial angle (MPTA) measurement

Choosing the appropriate paradigm is essential for ensuring operational reliability and interpretability in surgical decision support

Core transformer approaches (including Bidirectional Encoder Representations from Transformers [BERT]-style pretraining) established the basis for modern clinical natural language processing (NLP) and downstream instruction-tuned systems [26, 27]. When adapted to biomedical corpora and local clinical documentation, LLMs are particularly well-suited to tasks involving free text: extracting structured data from operative notes and radiology reports, summarizing multi-year EMR histories into problem-oriented notes, and generating patient-friendly explanations or discharge documentation at appropriate reading levels [16–18].

In orthopedics, the choice among these paradigms is largely dictated by input modality, data scale, and translational constraints. For tasks such as predicting transfusion, medication-related complications, or early readmission after arthroplasty, inputs are primarily structured (eg, age, body mass index (BMI), hemoglobin, comorbidities, and medications) [6, 7]. In this setting, ML models can be easier to calibrate and interpret for clinical use [7, 22]. By contrast, image-centric tasks are DL by design: fracture detection, OA grading, alignment measurement, and segmentation of cartilage or menisci require algorithms operating directly on pixel data [10, 13, 15]. Text and dialogue tasks are inherently linguistic, making LLMs the natural tool for supervised documentation and communication support [16–18].

Many clinically important problems are multimodal. A surgeon may want to combine preoperative EMR data with radiographs to predict revision risk, or integrate MRI-derived cartilage and meniscal features with patient-reported outcomes to forecast OA progression. Emerging approaches therefore fuse structured data, images, and text in unified architectures or orchestrate multiple specialized models. In practice, it is often wiser to begin with robust unimodal models (validated ML for structured data, well-validated CNNs for imaging, and constrained LLMs for text) and then integrate modalities iteratively.

### Study design and validation principles in orthopedic AI

Regardless of modeling paradigm, robust study design and validation are essential for trustworthy orthopedic AI. The starting point is a clear problem definition and clinically meaningful endpoint (eg, “Will this patient require transfusion within 72 h after total knee arthroplasty?” or “Is there a displaced femoral neck fracture on this radiograph?”) [6, 7].

Data handling and internal validation come next. At a minimum, datasets should be partitioned into training, validation, and test sets with separation at the patient level to prevent information leakage. Cross-validation can provide more stable performance estimates in modest-sized cohorts, but even strong internal metrics are insufficient to justify deployment.

Because practice patterns, imaging protocols, and patient populations vary across sites, external validation on independent institutions or time periods is widely regarded as a minimum requirement for translation [28, 29]. Prespecified out-of-distribution test sets (different hospitals, scanner vendors, or demographic subgroups) help reveal performance degradation under domain shift.

Evaluation metrics should extend beyond discrimination (how well a model separates patients with and without an outcome). Measures such as area under the receiver operating characteristic curve (AUROC), sensitivity, and specificity quantify separability but do not tell whether predicted probabilities are numerically accurate. Calibration addresses a practical clinical question: if the model reports a 20% transfusion risk, do roughly 1 in 5 similar patients actually receive transfusion? Miscalibration can lead to unnecessary preoperative crossmatching or false reassurance. Calibration plots and proper scoring rules (e.g., Brier score) summarize this agreement [30]. Decision-curve analysis translates model predictions into clinical value by estimating the “net benefit” of acting on predictions across plausible risk thresholds (e.g., the threshold at which you would intensify postoperative monitoring) compared with default strategies [31]. Reporting quality should also be transparent and standardized for prediction models, including adherence to established guidance where applicable [32].

Ultimately, the impact of orthopedic AI can only be established through prospective and pragmatic evaluation in real-world settings [28]. Designs comparing clinician performance with and without AI assistance, stepped-wedge rollouts, and carefully monitored pilot deployments provide richer evidence than retrospective validation alone. Evidence generation should include not only performance but also workflow burden, reliance patterns, and safety outcomes [28, 33].

## Recent research advances in orthopedic AI

### Patient outcome prediction

Predictive modeling in orthopedics has moved beyond proof-of-concept and now addresses perioperative problems with direct clinical relevance, particularly transfusion risk, early complications, and readmission. These models support preoperative planning, risk stratification, and shared decision-making when presented as calibrated risk estimates rather than stand-alone decisions [28].

**Fig. 2:**
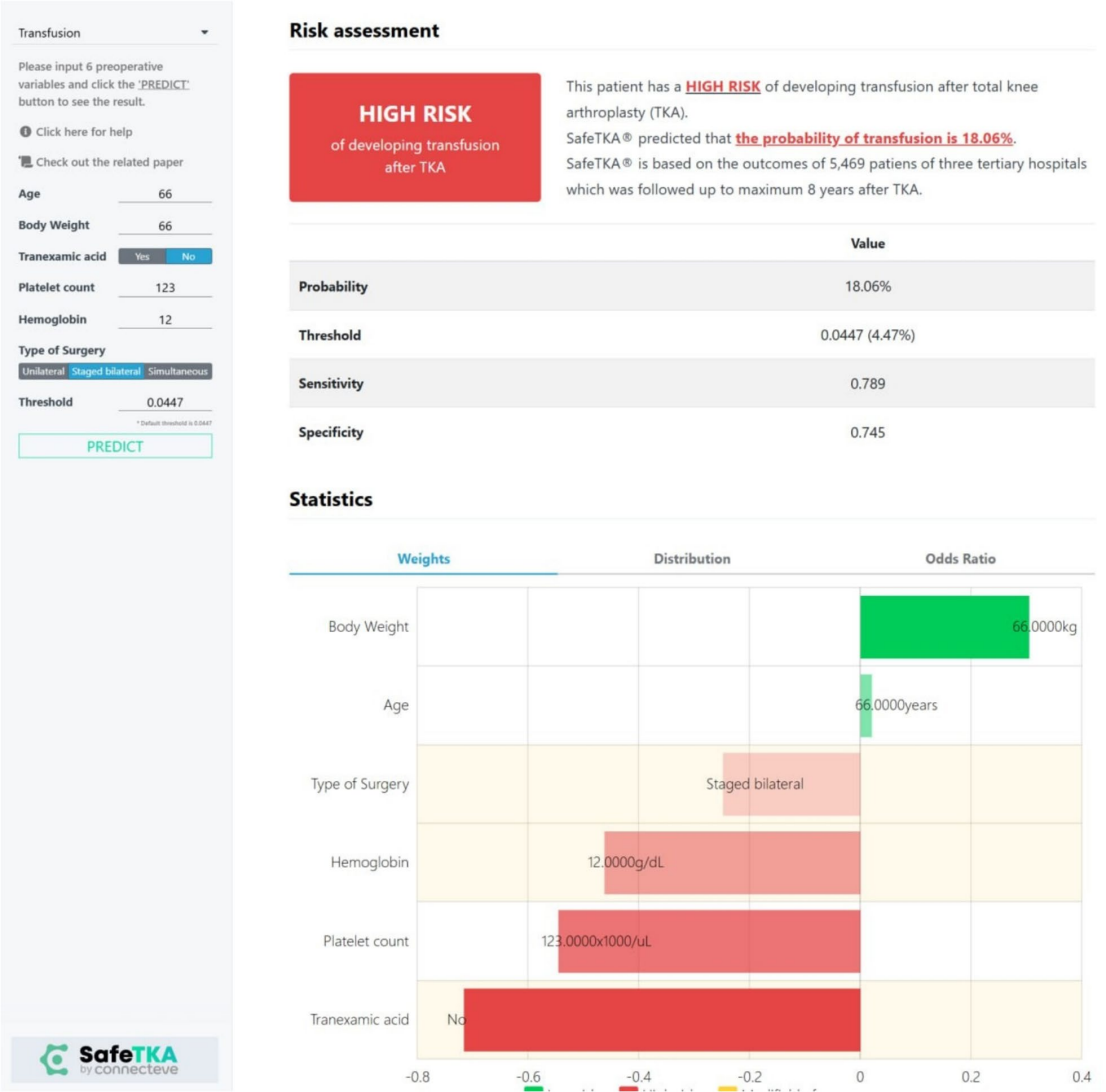
Example clinical interface for transfusion-risk prediction after total knee arthroplasty. The interface illustrates a patient-specific risk profile following total knee arthroplasty, integrating calibrated probability estimates with uncertainty indicators. Predictors (e.g., baseline hemoglobin, anticoagulant use, and comorbidity burden) are ranked by their relative contribution to the model’s output. A dedicated explanation panel provides a concise summary of the driving features to support transparent shared decision-making at the point of care. Screenshot reproduced with permission from Connecteve Inc. (SafeTKA web application) The underlying transfusion prediction model is described by Jo et al. [6]

For example, Jo et al. developed a machine learning model for postarthroplasty transfusion using Korean national insurance and single-center EMR data. The model achieved strong discrimination (internal AUROC 0.842, external AUROC 0.880) and outperformed conventional approaches, illustrating that routinely collected variables can enable robust risk prediction (Fig. 2) [6]. Similarly, outcome models have targeted postdischarge utilization. Using Australian registry and administrative data, Gould et al. compared ML models with logistic regression for 30-day readmission after primary arthroplasty; discrimination was modest but calibration supported pragmatic discharge planning when combined with clinical judgment [7].

Collectively, these studies suggest that ML-based outcome models are closest to translation where inputs are structured, end points are clearly defined, and predictions tie directly to concrete perioperative decisions.

### Medical image analysis

AI-powered image analysis is one of the most mature domains in orthopedic AI, driven by PACS-scale imaging availability and the performance of CNN-based methods [4]. Deep learning systems have demonstrated strong performance for fracture detection and classification, OA grading, and automated measurement tasks [13–15].

Zheng et al. validated a CNN to classify hip fractures on pelvic radiographs, achieving high diagnostic performance and demonstrating potential value for triage and training [13]. Multicenter evaluation for fracture detection at scale has also been demonstrated, including studies assessing AI assistance in appendicular skeletal fracture detection [34].

**Fig. 3:**
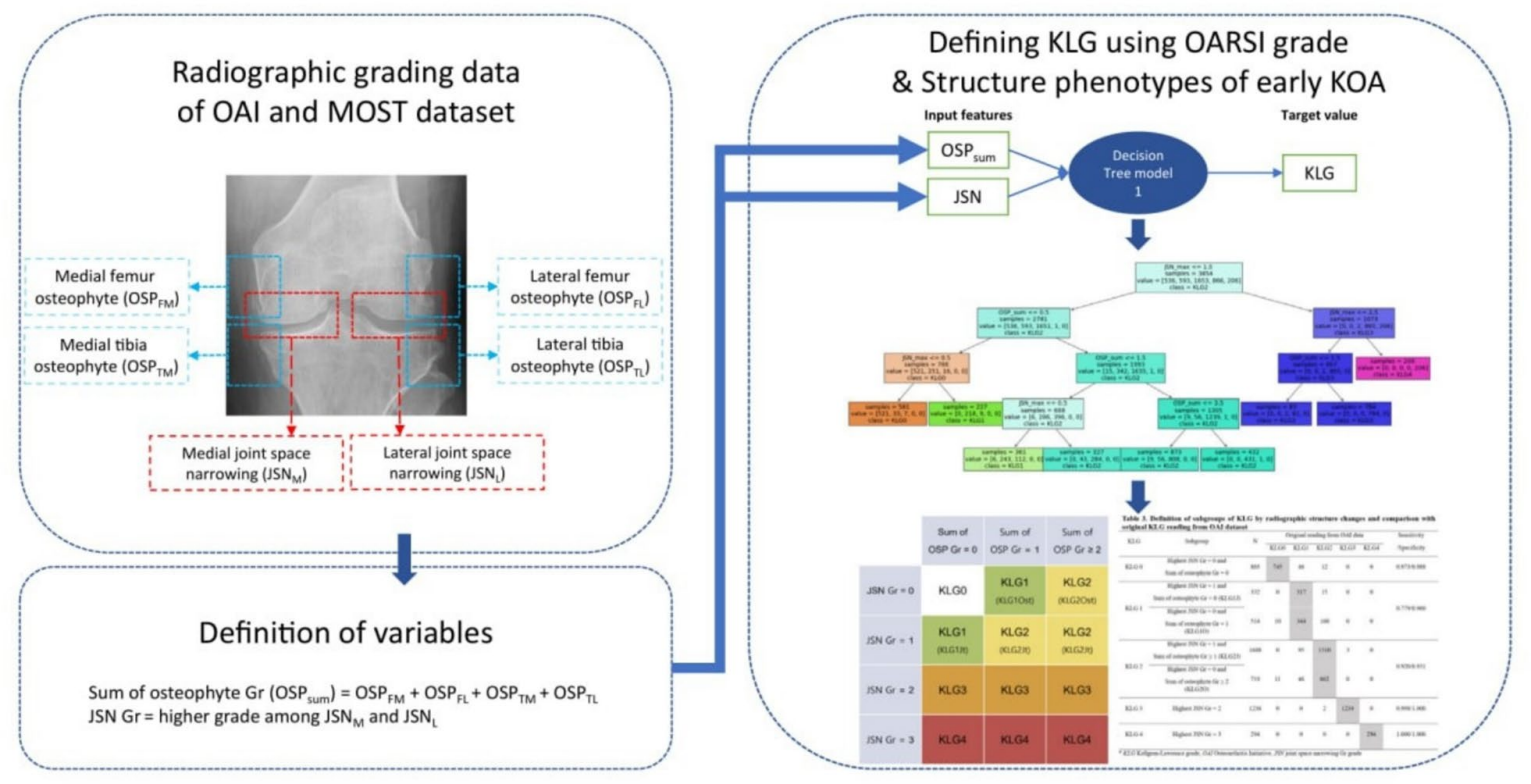
OARSI-feature-based decision logic for radiographic KL grading. This framework integrates semi-quantitative OARSI (Osteoarthritis Research Society International) features, including medial/lateral osteophyte (OSP) scores and joint-space narrowing (JSN) grades, into a reproducible classification pipeline. The logic demonstrates how structured radiographic features are mapped to KL categories (0–4), ensuring clinical interpretability and reducing inter-reader variability through standardized thresholds. Figure created by the authors, based on the OARSI-based KL grade definition framework described by Ko et al. [38]

Inter-reader variability remains a limitation in musculoskeletal radiology, particularly for Kellgren–Lawrence (KL) grading in knee osteoarthritis [35]. Deep learning classifiers have improved reproducibility and performance. External validation efforts for radiographic OA phenotyping show clinically meaningful agreement in independent datasets [36, 37]. Beyond composite KL grades, feature-focused pipelines can improve interpretability and reproducibility by mapping structured radiographic findings to KL categories (Fig. 3) [38].

**Fig. 4:**
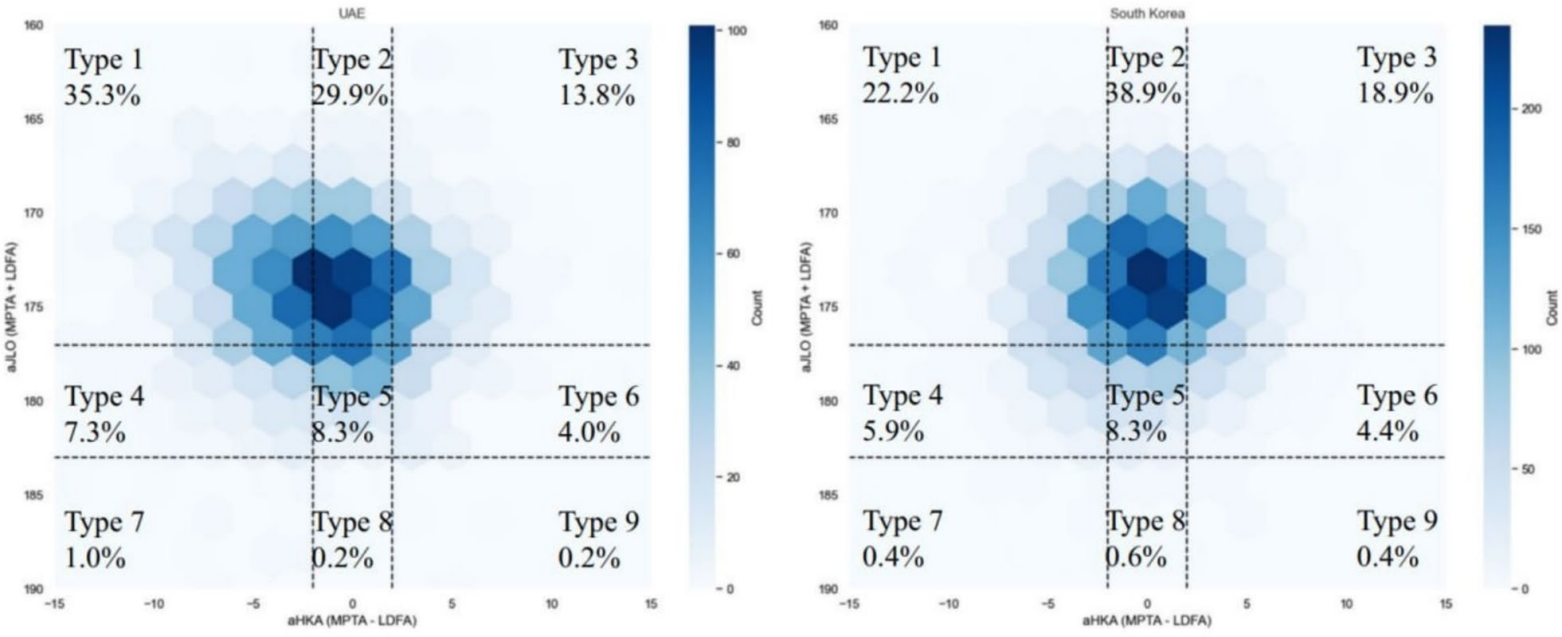
Distribution of coronal knee phenotypes across national cohorts using the CPAK framework. Utilizing the Coronal Plane Alignment of the Knee (CPAK) framework, the diagram depicts the relative frequency of CPAK Types 1–9 in two representative populations (e.g., UAE and South Korea). These cross-regional variations highlight the phenotypic diversity that must be accounted for in surgical planning and implant selection strategy. aHKA indicates arithmetic hip–knee–ankle angle; JLO, joint-line obliquity. Reproduced from Park et al. [41] under the Creative Commons Attribution 4.0 International License (CC BY 4.0)

As these imaging models approach potential deployment, the central question is generalizability across institutions and acquisition protocols. External validation is therefore a practical requirement, as performance can attenuate in independent settings under domain shift [28, 29]. Consistent with this, deep learning tools for posterior tibial slope measurement have demonstrated validation and external performance on independent radiographic cohorts [39, 40]. In Korea, AI pipelines have also been used to support multi-cohort alignment phenotyping, illustrating how standardized measurement automation can scale across heterogeneous health systems (Fig. 4) [41].

Building upward, teams are increasingly exploiting foundation-model strategies—large neural networks pretrained on broad, often unlabeled datasets and then adapted to specific tasks—and self-supervised learning to improve generalization while addressing privacy constraints and label scarcity [24, 25]. Quantitative MRI pipelines are also converging toward “digital phenotype” representations by combining multi-structure segmentation with standardized whole-joint scoring systems to summarize cartilage and meniscal status for downstream prediction [11, 42].

**Fig. 5:**
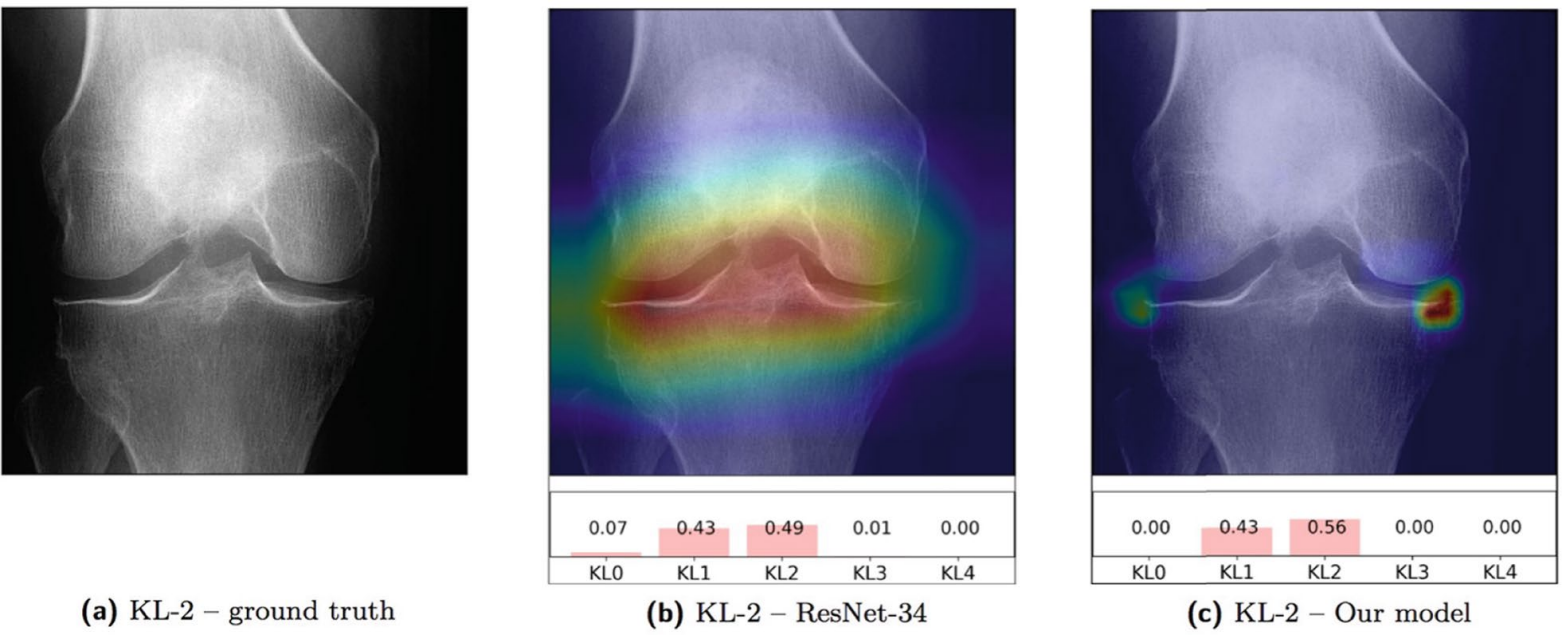
Chain-of-thought (CoT) rationale for automated Kellgren–Lawrence (KL) grading. **a** Input anteroposterior weight-bearing radiograph; **b** Standard Grad-CAM saliency showing nonspecific feature attribution; **c** Proposed model saliency localizing specific radiographic cues (e.g., medial compartment osteophytes and joint-space narrowing). The stepwise CoT rationale—mapping visual cues to intermediate findings and final KL-3 criteria—mirrors clinical reasoning to enhance user trust. The bottom panel illustrates the calibrated probability distribution across KL grades, providing a measure of model confidence. Adapted in style from Tiulpin et al., Scientific Reports 2018;8:1727 (CC BY 4.0), with modifications [8]

Finally, there is growing interest in interpretable, stepwise reasoning patterns (often described as chain-of-thought style) for imaging-driven decisions, where intermediate findings are surfaced alongside final outputs to mirror clinical logic (Fig. 5).

### Large language models (LLMs)

LLMs are evolving from generic text assistants into clinically oriented systems capable of documentation support, data extraction, and supervised dialogue-based clinical assistance [16–18]. Foundational transformer pretraining (eg, BERT-like paradigms) established the modern basis for clinical NLP, while newer large-scale systems support broader generation and summarization tasks [26, 27].

Recent reports describe conversational diagnostic AI systems that conduct structured history-taking and management discussions, demonstrating improvements in communication quality under blinded evaluation [16]. A randomized clinical trial (RCT) evaluation also indicates that LLM outputs can influence diagnostic reasoning in clinically meaningful ways—highlighting both potential benefit and the need for guardrails [17].

Orthopedics-specific evaluations have shown that high-performing general-purpose models can answer board-style questions with moderate accuracy, but still exhibit inconsistent and sometimes confident errors, supporting a supervised “copilot” role rather than autonomous clinical decision-making [43]. Related evidence shows variable performance on free-response reasoning exams and sensitivity to prompting, reinforcing the need to evaluate reliability under realistic clinical inputs [44].

Safety risks extend beyond hallucination to security vulnerabilities. Medical LLM outputs can be susceptible to prompt-injection-style attacks that distort advice under realistic dialogue conditions, underscoring the need for adversarial testing and deployment-time safeguards [45]. Real-world EMR-based summarization studies show promise but also emphasize monitoring, auditability, and clear intended-use boundaries [46]. Expert-level medical question-answering efforts further highlight the value of retrieval grounding and constrained outputs for clinical reliability [47].

### Translational maturity across orthopedic AI domains: where is it happening first?

Across current applications, orthopedic AI appears closest to routine clinical use in two areas: structured outcome prediction and imaging-based measurement automation. Risk models built from EMR and registry variables can be integrated as calibrated risk calculators when supported by appropriate evaluation frameworks [28]. Imaging AI achieves reader-level reproducibility for fracture detection, OA grading, and alignment measurements, enabling scalable phenotyping and workflow support [14, 15]. By contrast, LLMs remain earlier-stage in orthopedics and are currently best suited to supervised copilot roles in low-risk tasks, where value is measured by documentation efficiency, communication clarity, and robust safety guardrails rather than benchmark accuracy alone [16–18].

## Cross-cutting challenges on the path to clinical translation

### Data heterogeneity and generalizability

Orthopedic AI models can be brittle because they are trained on single-institution datasets with narrow demographic and technical profiles, leading to attenuation when evaluated outside the development setting. This undermines reliability across different case-mix, workflows, and acquisition environments and contributes to overly optimistic claims when studies emphasize internal validation without adequately characterizing heterogeneity. Translation-oriented evaluation increasingly requires multi-institutional cohorts and heterogeneous test environments that reflect real-world variation [28, 29].

Beyond data volume, a persistent bottleneck is labeling and outcome definition: high-fidelity annotations require scarce expert time, and inconsistency in labels and reporting practices can degrade generalizability. Large-scale datasets that explicitly encode uncertainty and label provenance illustrate both the opportunity and the complexity of scalable labeling [48].

### Algorithmic opacity and explainability

The “black box” nature of many deep learning systems remains a barrier to adoption, particularly in orthopedics where surgical decisions and implant choices are difficult to reverse. Systematic evaluations in clinical AI highlight that design, reporting, and claims often outpace deployment-grade evidence, reinforcing the need for transparency about limitations and failure modes [29].

Equally important is uncertainty and reliability: models should provide well-characterized performance with appropriate confidence bounds and, when used for decision support, should be assessed using frameworks that go beyond discrimination to include calibration and clinical usefulness [30, 31]. In deployment-facing contexts, clear documentation of intended use, assumptions, and boundaries is essential so clinicians recognize when an algorithm is extrapolating beyond its familiar domain [28].

### Legal, regulatory, and ethical governance

Regulatory frameworks for AI in medicine are shifting toward life-cycle oversight, emphasizing real-world evaluation, reporting rigor, and postdeployment risk management [49, 50]. Governance frameworks for enterprise-scale healthcare AI emphasize risk mitigation, versioning, and monitoring to maintain safety over time [20]. Reporting standards for AI interventions (e.g., CONSORT-AI: Consolidated Standards of Reporting Trials–Artificial Intelligence) and early-stage evaluation guidance (e.g., DECIDE-AI: Developmental and Exploratory Clinical Investigations of Decision support systems driven by Artificial Intelligence) clarify what should be documented—intended use, workflow context, human–AI interaction, and clinically meaningful outcomes—before claims of benefit are considered credible [28, 33].

Ethical considerations are equally central. Models trained on skewed datasets can encode and amplify disparities in access and outcomes; therefore, subgroup performance audits and fairness-aware evaluation should be routine, with mitigation plans defined in advance [51, 52]. Accountability should be clearly delineated across developers, vendors, and healthcare organizations, especially when AI is embedded into clinical workflows where responsibility might otherwise be ambiguously shifted to end users [20].

### Workflow integration and implementation science

Many accurate models fail at the point of use because they do not fit real clinical workflows. Translation depends on interoperability with EMR/PACS environments, careful user-experience design, and minimizing added cognitive load in clinic, ward, and operating-room settings. Evidence frameworks for early-stage clinical evaluation emphasize measuring not only accuracy but also workflow impact, including time, edit burden, handoff quality, reliance patterns, and safety signals [28].

Accordingly, orthopedic AI should be evaluated in staged, deployment-relevant designs (eg, silent-mode runs and prospective usability-focused studies) where outcomes include documentation/process metrics and patient-centered outcomes when appropriate. Decision support tools should be treated as sociotechnical systems rather than standalone algorithms, aligning governance and implementation with real-world risk mitigation [20, 28].

### Practical considerations and future directions for orthopedic surgeons

From the orthopedic surgeon’s perspective, the near-term opportunity is to treat AI as a supervised “copilot” that improves consistency and efficiency in tasks already embedded in daily care (e.g., standardized radiographic measurements, triage-level fracture detection, and calibrated perioperative risk calculators). Successful adoption will depend less on selecting the most complex model and more on defining an intended use with clear boundaries, training users to recognize failure modes, and minimizing workflow friction within PACS/EMR environments.

Practically, we anticipate that surgeon-led implementation will increasingly resemble other quality-improvement initiatives: start with a silent-mode pilot to quantify local performance and drift; agree on actionable thresholds and escalation pathways; monitor calibration and subgroup performance over time; and document human–AI interaction to avoid automation bias. Looking forward, multimodal “digital twin” concepts may enable patient-specific trajectories that combine imaging phenotypes, structured EMR variables, and intraoperative data, but these systems will only be credible if they demonstrate net benefit in prospective evaluations and remain accountable through life-cycle governance.

## Conclusion: from experimental benchmarks to clinical accountability

The evolution of orthopedic AI has reached an inflection point at which emphasis must shift from retrospective benchmark performance to clinical utility in practice. Internal performance alone is insufficient when models are poorly calibrated, do not quantify uncertainty, or degrade under domain shift across scanners, protocols, and patient populations. External validation across independent settings should be regarded as a minimum requirement for clinical readiness, alongside calibration assessment and prespecified reporting of failure modes [28, 29].

A second direction is the transition from isolated, task-specific models toward multimodal convergence. Specialty-adapted foundation strategies may enable longitudinal patient representations by integrating structured EMR variables, imaging phenotypes, and perioperative data into unified trajectories [24, 25]. Realizing this potential will depend as much on implementation as on algorithmic development: decision support must be delivered within native PACS/EMR workflows with minimal added burden and evaluated prospectively using outcomes that reflect net clinical benefit [28].

Ultimately, orthopedic AI should be judged by transparency and accountability rather than technical novelty. This requires life-cycle governance—versioning and change control, drift monitoring, and routine performance audits—to maintain safety over time. Prioritizing reliability, governance, and workflow fit over retrospective metrics provides the most direct path to meaningful adoption of AI in orthopedic practice [20].

## Data Availability

Data sharing is not applicable to this article as no datasets were generated or analyzed during the current study.

## Abbreviations

aHKA: Arithmetic hip–knee–ankle angle
AI: Artificial intelligence
AUROC: Area under the receiver operating characteristic curve
BERT: Bidirectional encoder representations from transformers
BMI: Body mass index
CC BY 4.0: Creative commons attribution 4.0 international license
CNN: Convolutional neural network
CONSORT-AI: Consolidated standards of reporting trials–artificial intelligence
CoT: Chain of thought
CPAK: Coronal plane alignment of the knee
CPU: Central processing unit
CT: Computed tomography
DECIDE-AI: Developmental and exploratory clinical investigations of decision support systems driven by artificial intelligence
DICOM: Digital imaging and communications in medicine
DL: Deep learning
EMR: Electronic medical record
GPU: Graphics processing unit
Grad-CAM: Gradient-weighted class activation mapping
HKAA: Hip–knee–ankle angle
ICU: Intensive care unit
JLO: Joint-line obliquity
JSN: Joint-space narrowing
KHIDI: Korea Health Industry Development Institute
KL: Kellgren–Lawrence
LLM: Large language model
MIMIC-IV: Medical information mart for intensive care IV
ML: Machine learning
MPTA: Medial proximal tibial angle
MRI: Magnetic resonance imaging
NLP: Natural language processing
OA: Osteoarthritis
OARSI: Osteoarthritis Research Society International
OSP: Osteophyte
PACS: Picture archiving and communication system
RCT: Randomized controlled trial
SHAP: Shapley additive explanations
TPU: Tensor processing unit
U-Net: U-shaped network
ViT: Vision transformer
XAI: Explainable artificial intelligence
XGBoost: EXtreme gradient boosting
